# Integrated Nanosecond Pulse Irreversible Electroporation (INSPIRE): Impact of Exposed Electrode Length on Ablation Geometry in an In Vivo Liver Model

**DOI:** 10.3390/cancers17172891

**Published:** 2025-09-02

**Authors:** Jordan A. Fong, Logan Reeg, Jewels Darrow, Robert H. Williamson, Anna Riordan, Alexia K. Cash, Max Beecroft, Callie A. Fogle, Kyle G. Mathews, Nathan C. Nelson, Alina C. Iuga, David A. Gerber, Michael B. Sano

**Affiliations:** 1 Gradient Medical Inc., Raleigh, NC 27607, USA; 2 College of Agriculture and Life Sciences, North Carolina State University, Raleigh, NC 27695, USA; 3 College of Veterinary Medicine, North Carolina State University, Raleigh, NC 27607, USAmcbeecro@ncsu.edu (M.B.);; 4 Lampe Joint Department of Biomedical Engineering, North Carolina State University, Raleigh, NC 27695, USA; 5 Department of Pathology and Laboratory Medicine, School of Medicine, University of North Carolina, Chapel Hill, NC 27599, USA; 6 Department of Surgery, College of Medicine, University of Cincinnati, Cincinnati, OH 45267, USA

**Keywords:** INSPIRE, IRE, pulsed field ablation, inoperable tumors, focal ablation

## Abstract

**Objectives:** There is a critical need for effective focal therapies for patients with inoperable or anatomically complex tumors where conventional ablation techniques pose high risk or are ineffective. Integrated Nanosecond Pulsed Irreversible Electroporation (INSPIRE) is a novel non-thermal ablation modality which uses real time temperature feedback during pulse delivery to safely treat tumors near critical structures. This study evaluated the impact of exposed electrode length on ablation zone size, reproducibility, and cardiac safety in a large animal model. **Methods:** INSPIRE treatments were performed in an in vivo healthy porcine liver model. All treatments administered 6000 V 1000 ns pulses with a 45 °C temperature set point. Treatments were administered percutaneously via an electrode and grounding pad approach using an internally cooled electrode applicator. The exposed electrode region at the distal end of the applicator was set to either 0.5, 1.0, 1.5, or 2.0 cm. Ablation zones were assessed via ultrasound, contrast-enhanced CT, and gross pathology one week post-treatment. Cardiac safety was evaluated by measuring pre- and post-treatment serum Troponin levels. **Results:** All treatments were completed without adverse events. Troponin levels remained stable (pre: 0.249 ng/mL; post: 0.224 ng/mL), indicating no measurable cardiac injury. The 1.5 cm exposure length produced the largest and most consistent ablation volumes, with a mean volume of 12.8 ± 2.6 cm^3^ and average dimensions of 3.7 × 2.7 cm in under 6 min. Increasing exposure length beyond 1.5 cm introduced greater variability and reduced treatment volumes. **Conclusions:** INSPIRE enables safe, large-volume, single-applicator ablation without a need for electrical pulse synchronization with R wave in cardiac rhythm. The 1.5 cm exposure length offers optimal balance between energy delivery and treatment consistency. These findings support further clinical investigation of INSPIRE for non-thermal ablation of inoperable tumors.

## Simple Summary

Irreversible electroporation (IRE) is increasingly used to treat tumors in anatomically complex locations. However, clinical delivery can be challenging due to the potential for cardiac complications, intense muscle stimulation, and the need for multiple needle electrodes placed in parallel with precision under ultrasound guidance. This study evaluated INSPIRE, a next-generation IRE platform using bipolar pulse waveforms (1000 ns) and high-voltage (6000 V) pulses with real-time temperature control (45 °C). Using a simplified single-electrode and grounding pad approach, we found that a 1.5 cm electrode exposure length produced the most consistent and voluminous ablation zones (up to 12.8 cm^3^ in <6 min). Treatments were visualized well by contrast-enhanced CT and intraoperative ultrasound, and cardiac safety was confirmed by stable Troponin levels. These findings suggest that optimizing applicator geometry in INSPIRE can streamline workflow, enhance reproducibility, and expand safe access to non-thermal ablation in challenging clinical scenarios.

## 1. Introduction

Minimally invasive ablation therapies using imaging to guide applicator placement via percutaneous or laparoscopic approaches have become increasingly common in clinical oncology practice [1,2,3]. Microwave ablation (MWA), radio-frequency ablation (RFA), and cryo-ablation (CA) are well established focal ablation technologies which rapidly destroy tumor tissue via the rapid heating or cooling of the target tissue. However, these approaches are contraindicated in scenarios where there is increased risk of thermal damage to sensitive nearby structures [4] or when the presence of heat sinks reduces ablation efficacy [5,6].

**Figure 1:**
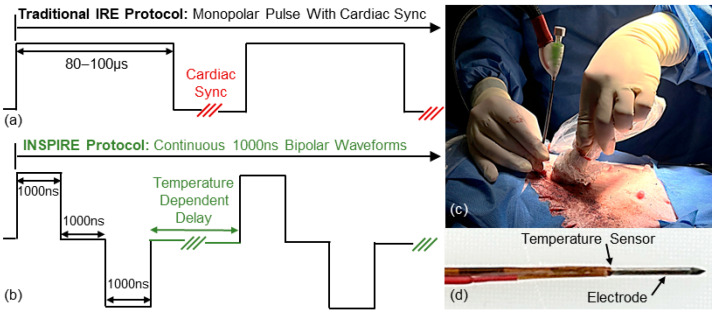
INSPIRE enables delivery via a single applicator and grounding pad approach. (**a**) Traditional monopolar 80–100 µs pulse waveform administered with cardiac synchronization in NK-IRE treatments. (**b**) INSPIRE waveforms are continuously delivered with a variable delay between sequential waveforms based on real time temperature feedback. (**c**) INSPIRE waveforms administered percutaneously under ultrasound guidance in in vivo liver with a (**d**) single temperature sensing applicator and grounding pad configuration.

Pulsed field ablation (PFA) is an emerging non-thermal focal ablation approach which uses high-voltage, short-duration electrical pulses to destabilize the cellular membrane and induce cell death through a process known as irreversible electroporation (IRE) [7]. The Angio Dynamics NanoKnife (NK-IRE), the first FDA-cleared ablation device, administers a series of 90–100× electrical pulses each 80–100 µs in duration (Figure 1a). NK-IRE has been evaluated in over 50 clinical trials [8] for the treatment of pancreatic [9,10,11], prostate [12,13,14], kidney [15,16], and liver [17,18,19,20] tumors.

While NK-IRE has been demonstrated as a safe and effective therapy, there are significant challenges which may limit broad clinical adoption of the technique. NK-IRE requires the precise placement of multiple (2–6) electrodes in parallel surrounding the tumor. Energy is then administered with cardiac synchronization [21] between individual probe-pairs to mitigate potential cardiac complications [21,22]. Intense muscle stimulation requires the use of intraoperative neuromuscular paralytics [23] and breakthrough muscle stimulation has been reported in some cases despite achieving complete medicinal neuromuscular block [24]. Limitations in NK-IRE device power outputs (3000 V, 50 A) result in relatively small treatment zones. To overcome this, applicators are typically placed at the distal aspect of a tumor followed by multiple treat-then-retract cycles resulting in prolonged needle placement times (approximately 20 min) and energy delivery times averaging approximately 35 min for the treatment 3 cm tumors [25]. Finally, while NK-IRE is considered a non-thermal ablation modality, resistive losses in the tissue result in Joule heating [26,27]. A lack of standardized treatment protocols [10] can lead to scenarios where extreme heating and thermal injury occur [28].

To address challenges with NK-IRE, a number of approaches have been proposed including the use of micro- to nanosecond pulses [29,30,31]. High-frequency IRE (H-FIRE) administers a rapid burst of alternating polarity electrical pulses lasting 50–100 µs mimicking the NK-IRE waveform [32]. This burst waveform is then repeated 100× with cardiac synchronization [33]. Clinical H-FIRE systems (Aliya PEF) alleviate muscle stimulation, but produce sub-centimeter ablation zones [34] and require multiple hours of energy delivery to treat nominal tumor volumes [33]. Clinical nanosecond pulsed electric field (nsPEF) treatments administer a series of 800 × 300 ns pulses with cardiac synchronization via 2–16 parallel electrodes inserted into the tumor [35]. While a potentially promising approach for inducing an anti-tumor immune response [36,37], nsPEF requires extreme voltages (15–30 kV) and a large number of applicators to achieve therapeutic effect [38]. While promising PFA approaches, intense muscle stimulation (NK-IRE), the need for numerous overlapping treatments (H-FIRE), extreme electric field strengths applied via large electrode arrays (nsPEF), and the potential to induce inadvertent thermal injury (all current clinical PEF systems) add significant complexity for the treatment of inoperable tumors which often form near critical structures.

Integrated Nanosecond Pulsed Irreversible Electroporation (INSPIRE) aims to address these challenges. To reduce muscle stimulation, INSPIRE administers an alternating polarity waveform consisting of a paired positive and negative pulses generally in the range of 250 ns to 2000 ns [39,40] (Figure 1b). Significant reductions in muscle stimulation [30] enable the use of a single applicator and grounding pad approach [32,41,42] similar to those employed in RFA to produce predictable spherical ablation zones [43] that may help to reduce pre-treatment planning complexity. INSPIRE waveforms are administered continuously throughout the cardiac cycle using a temperature control algorithm [44] to dynamically adjust the delay between sequential waveforms to achieve and maintain a target temperature within the tissue [39,40]. This active temperature control is supplemented with electrode cooling to dramatically reduce treatment times [45].

INSPIRE treatments have been shown to be safe and effective for the treatment of spontaneous tumors in veterinary models [39,46] and ongoing large animal studies are being conducted to evaluate the effect of pulse width, temperature setpoints, and electrical dosing on treatment outcomes. This study aimed to demonstrate the safety and efficacy of high voltage (6000 V) INSPIRE treatments in healthy liver parenchyma and determine the effect of the exposed electrode length (0.5–2.0 cm) on ablation zone size and shape. Ablation volumes were found to increase sequentially as the exposed electrode length was increased from 0.5 to 1.5 cm followed by a decrease in overall volume for the 2.0 cm configuration. All procedures were completed without adverse events and serum troponin levels, a marker of cardiac safety, did not significantly increase from baseline. These results indicate that INSPIRE may be a safe and effective method for rapid tumor ablation.

## 2. Materials and Methods

### 2.1. In Vivo Porcine Procedure

An in vivo study was completed in female Yorkshire pigs (45–55 kg, Palmetto Research Swine) to evaluate the effect of exposure length on INSPIRE ablation zone geometry. All studies were conducted under an approved IACUC protocol at the NCSU College of Veterinary Medicine. All procedures were performed percutaneously via ultrasound guidance to place the applicator in healthy liver parenchyma (Figure 1c). A custom pulse generator capable of 6000 V, 200 A outputs with a custom software package that enabled real time temperature control over waveform delivery was used to administer all treatments. A series of custom internally cooled electrode applicators were developed containing a fiberoptic temperature sensor at the insulator-electrode-tissue interface (Figure 1d). The applicator body was created from a 20 cm 18-gauge stainless-steel needle with a trocar tip. The needle was covered in a polyimide sheathing that left the distal end exposed. The length of exposed electrode on each applicator was fixed to either 0.5 cm, 1.0 cm, 1.5 cm or 2.0 cm (Table 1). The proximal end of the applicator houses cooling lines which circulated water through the device via a peristaltic pump at a rate of 10 mL/min.

Animals were fasted for 12 h then sedated under general anesthesia using 0.022–0.044 mL/kg TKX (50 mg/mL each of tiletamine, zolazepam, ketamine xylazine) solution and maintained with 1–3% isoflurane inhalation in a sterile operating suite. Neuromuscular stimulation was managed with 1–1.5 mg/kg IV injection dose of Rocuronium and a continuous infusion of 0.1–0.6 mg/kg throughout the treatments. Boluses of 0.75 mg/kg were administered between treatments if necessary to maintain a 0 out of 4 twitch score in accordance with a Train of Four monitor (Stimpod NMS450X, Xavant Technology Ltd., Silverton, Pretoria, South Africa). Heart rate, blood pressure, respiration, and temperatures were monitored continuously during treatment. Samples of blood were drawn prior to the beginning of treatment, immediately post-treatment, and then pre-euthanasia. Standard procedures were followed for a complete blood count and standard blood chemistry. In addition, troponin levels were quantified as a measure of cardiac injury using an Immulite 2000 Troponin I assay.

All treatments were administered via a percutaneous approach under ultrasound guidance. Under the same anesthetic event, three treatments were administered in discrete regions of the healthy liver parenchyma. A singular electrode applicator was inserted into the liver and a grounding pad on the lower back acted as the electrical return path. All treatments were administered with the same INSPIRE parameters (Voltage: 6000 V, Dose: 0.02 s, Temperature: 45 °C). For all treatments, a symmetric alternating polarity waveform consisting of 1000 ns positive and negative pulses separated by 1000 ns was administered. Electrical dose in INSPIRE is calculated as the sum of all pulse durations delivered. For example, a 0.02 s dose is achieved by administering 20,000 × 1000 ns pulses. This is approximately twice the dose of a typical NK-IRE protocol which administers 80–100 × 100 µs pulses (100 × 100 µs = 0.01 s).

This study was not blinded. The treatments were selected in a sequential order with three of the same exposed electrode lengths being administered into the same liver. Any treatments that bisected liver lobes or extended past the liver capsule were excluded from analysis. A five to nine replicates were included for each group (see Table 1 for details).

Post-treatment the animals received a fentanyl patch as prophylactic pain management following the procedure. They were monitored twice daily for the development of complications. A follow-up CT scan one week post treatment was conducted prior to euthanasia to measure the treatment zones. Following the CT scan the livers were imaged via ultrasound to identify the treatment zones via a second modality. The pigs were then humanely euthanized and the livers were resected. Gross pathology sections were obtained by palpating the liver to identify treatment sites and carefully sectioning along the midline of the site. Slicer 3D (V5.6.0) was used to recreate treatment zones from CT images and determine the volume for each ablation. Length and two perpendicular width measurements were collected for each treatment to enable assessment of sphericity of each treatment zone.

Statistical analyses were performed using Microsoft Excel. Group comparisons were assessed using a two-tailed Welch’s *t*-test assuming unequal variance, with statistical significance defined as *p* < 0.05. Data for treatment times, lethal electric field thresholds as well as CT measurements of treatment volumes, lengths, and widths are presented as mean ± standard deviation.

### 2.2. Numerical Modeling

Simulations predicting treatment zones were conducted using COMSOL Multiphysics (V 6.2, COMSOL Inc., Los Altos, CA, USA) via the electromagnetic heating, fluid dynamics and non-isothermal flow modules. These models used heat transfer and electric current modules to calculate electric field driven heating in a time dependent model. The time dependent electric field distribution was calculated as follows:

∇·J= 0

J=σE

E=−∇V

where **J** the current density [A/m^2^], **E** is the electric field [V/m], *V* is the applied electric potential [V], and σ is electrical conductivity [S/m]. Electromagnetic heating was calculated as follows:

$$Q_{e}=R\left(t\right)(J\cdot E)$$

where R(t) is the instantaneous energy delivery rate [µs/s] determined by the temperature feedback algorithm. The baseline electrical conductivity of the tissue (0.4 [S/m]) was selected from the IT IS Foundation database for liver at 1 MHz. Heat transfer in Solids module was calculated in a time dependent model as follows:

$$\rho C_{p}\frac{\delta T}{\delta t}\;-k\nabla \cdot \left(\nabla T\right)=Q_{e}$$

where ρ is the density [kg/m^3^], C_p_ is the heat capacity [J/kg·K], k is the thermal conductivity [W/(m·K)], and T is the local temperature [K].

**Figure 2:**
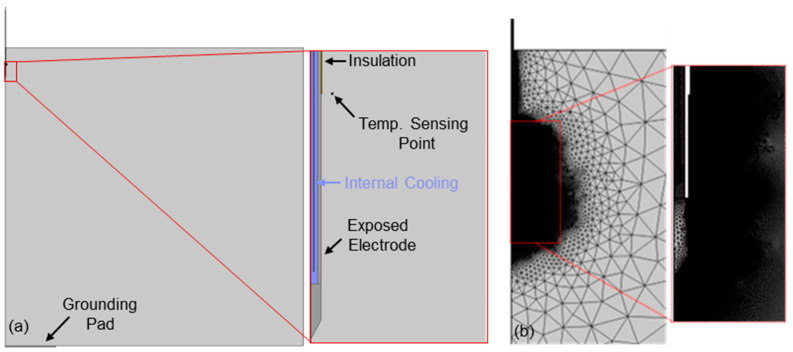
Numerical analysis used to estimate lethal electric field thresholds. (**a**) Simulations were conducted in a 2D axial-symmetric geometry to determine the minimum electric field strength associated with in vivo treatment margins. (**b**) The final refined mesh contained approximately 842,183 elements.

The abdomen and liver were modeled as a 45 cm long, 45 cm radial cylinder in a 2D axial-symmetric geometry (Figure 2a). The grounding pad was modeled as a 7.6 cm radius, 0.2 cm tall cylinder on the bottom of the tissue domain. The applicator was modeled as a 0.08255 cm radius tube with a 0.0228 cm internal channel to simulate the internally cooled electrode. The outer tube was encased in a 0.02032 cm layer of insulation from the proximal end down to the exposed electrode region at the distal end. A point probe was placed at the intersection of the insulation and exposed electrode.

Fluid domains were initially meshed with a maximum element size of 100 µm while all other domains had a maximum element size of 5.14 mm. Following an initial steady state simulation to calculate a baseline electric field distribution, two rounds of adaptive meshing were then conducted using an electric field-based error expression (ec.normE > 250 [V/cm]) to refine the mesh in regions with high electric field strength near the exposed electrode (Figure 2b). Individual meshes were created for the 0.5 cm, 1.0 cm, 1.5 cm, and 2.0 cm electrode geometries which contained between 269,493 and 842,183 elements requiring approximately 14–22 min to solve for each geometry on an AMD 64-core 5995WX processor with 512 GB of RAM.

Lethal electric field strengths were calculated by matching the experimental CT reconstruction volumes to simulation. The volume of tissue exposed to a specific electric field intensity was calculated via a surface integration with the expression ec.normE > E_set_ where E_set_ was equal to 200–2000 V/cm in 10 V/cm increments. The experimental volumes and the calculated simulation volumes were then matched to determine the lethal electric field for each experimental ablation.

## 3. Results

### 3.1. In Vivo Results

**Figure 3:**
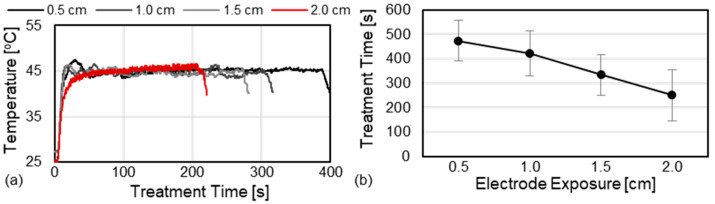
INSPIRE uses active temperature control to prevent thermal injury. (**a**) Representative temperature profiles and (**b**) average treatment times as a function of electrode exposure length. Error bars represent one standard deviation from the mean.

All treatments were successfully completed without complications and no muscle stimulation was observed with the 6000 V INSPIRE protocol. Treatment temperatures increased from baseline (approximately 25 °C) to 45 °C within the first 30–60 s of each treatment (Figure 3a). During this period, the temperature control algorithm gradually increased the delay between sequential waveforms and stable tissue temperatures of 45 °C were achieved with all exposed electrode lengths. Shorter exposed electrode lengths resulted in longer treatment times with 0.5 cm exposures requring 473 ± 83 s (7.9 min) and 2.0 cm exposures requring 250 ± 105 s (4.2 min) to administer the prescribed 0.02 s dose (10,000 waveforms each containing one positive and one negative pulse) (Figure 3b). This followed a linear trend (R^2^ = 0.98) and was likely due to an increase in the distance between the temperature sensor and the region of maximal heating at the electrode tip resulting in lower measured temperatures and faster algorithmic waveform delivery.

**Figure 4:**
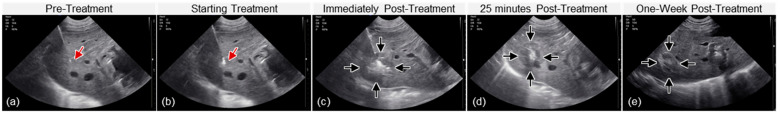
INSPIRE treatments are visible on intraoperative ultrasound. Treatment administered near blood vessels (**a**) pre-treatment, (**b**) during beginning of treatment, (**c**) immediately post-treatment, (**d**) post-treatment, and (**e**) one-week post-treatment. Red arrows indicate electrode placement. Black arrows indicate visible ablation margins.

Ablation zones were visible on ultrasound intraprocedurally (Figure 4). A hyperechoic region corresponding to the exposed electrode was visible immediately upon initiation of the treatment potentially due to gas bubble formation (Figure 4b). This hyperechoic region expanded throughout the treatment and was gradually replaced by a contrasting hypoechoic region (Figure 4c) post treatment with increasing contrast between the treated region and healthy liver in the intraoperative period (Figure 4d). One week post-treatment, the ablations were clearly visible on ultrasound typically characterized by a hyperechoic ring surrounding a contrasting hypoechoic region (Figure 4e).

**Figure 5:**
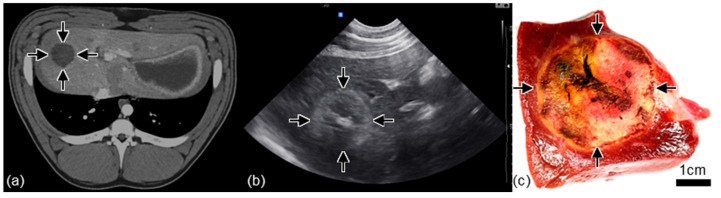
Spherical ablation zones visible on CT and ultrasound match gross pathology. One-week post-treatment imaging of the same ablation site, created with a 1 cm electrode exposure. (**a**) The ablation was visible on contrast-enhanced CT as a hypodense region. (**b**) On ultrasound the treatment sites appeared as a hyperechoic region. Occasionally, the location of the applicator appeared as a bright hyperechoic region in the center of the treatment zone. (**c**) The ablations were clearly visible on gross tissue sections and appeared as a region of pale or discolored tissue surrounded by a fibrous capsule. Arrows indicate the margins of the treatment zones.

**Figure 6:**
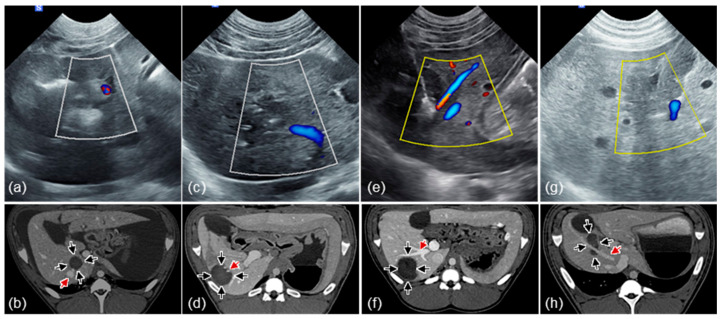
Preservation of blood vessels adjacent to and within treatment zones. Doppler ultrasound (immediately post) and contrast-enhanced CT (one week post) images identifying perfused blood vessels within and proximal to the treatment sites. Representative images of treatments administered with a (**a**,**b**) 0.5 cm, (**c**,**d**) 1.0 cm, (**e**,**f**) 1.5 cm and (**g**,**h**) 2.0 cm electrode exposure length. Black arrows indicate approximate treatment margins and red arrows indicate blood vessels.

One week post-treatment, the ablations were similarly visible on contrast-enhanced CT as a radiolucent region (Figure 5a) corresponding to the hypoechoic region visible on ultrasound (Figure 5b). On ultrasound, the ablations occasionally contained a enhanced hyperechoic region corresponding to the location of the applicator (Figure 5b). These imaging changes were relfected in treatment zones observed in gross tissue sections (Figure 5c). Blood vessels that were near or in the treatment zone were generally well perfused immediately following and one week post treatment per Doppler ultrasound and CT imaging (Figure 6).

**Figure 7:**
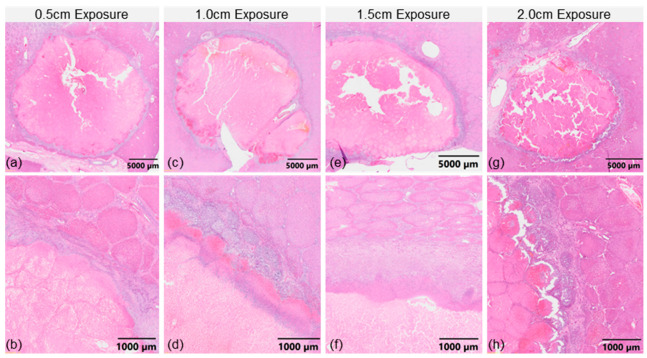
Sub-millimeter Transition Between Live and Dead Tissue. Hematoxylin and eosin stained histological slides of from INSPIRE treatments with (**a**,**b**) 0.5 cm, (**c**,**d**) 1.0 cm, (**e**,**f**) 1.5 cm, and (**g**,**h**) 2.0 cm electrode exposures.

Following the excision of the livers, the treatment zones were readily found via palpitation as the regions had an increased stiffness in comparison to the healthy liver parenchyma. The bisected treatment zones appeared heterogeneous with a narrow pink capsule surrounding the treated tissue (Figure 5c). There was a clear transition between normal and treated tissue on H&E stained tissue samples (Figure 7). Inside the treatment zone there was coagulative-type necrosis with preserved cellular outlines, hypereosinophilic cytoplasm and karyolysis. Towards the periphery of the treatment zone, foci of cells with nuclear karyorrhexis, incomplete karyolysis, infiltrating degenerative neutrophils and congestion were observed. On the border of the treatment zone there was a capsule-like reaction consisting of fibrosis with reactive fibroblastic proliferation, chronic lymphoplasmacytic inflammation with scattered neutrophils and eosinophils. There was evidence of a foreign-body type multinucleated giant cell reaction with microcalcifications and iatrogenic black pigment deposition. External to the fibrous capsule, there were patchy parenchymal changes consisting of portal vascular and sinusoidal dilation and congestion. Eosinophilic hepatocytic cytoplasmic changes, reactive endothelial cells, and occasional vascular thrombi were also seen.

No statistically significant changes in Troponin levels were found between pre-treatment (0.249 ± 0.076 ng/mL) and post-treatment (0.224 ± 0.047 ng/mL) or one-week post-treatment (0.202 ± 0.005 ng/mL). The maximum values for these respective groups were 0.446, 0.331, and 0.218 ng/mL.

**Figure 8:**
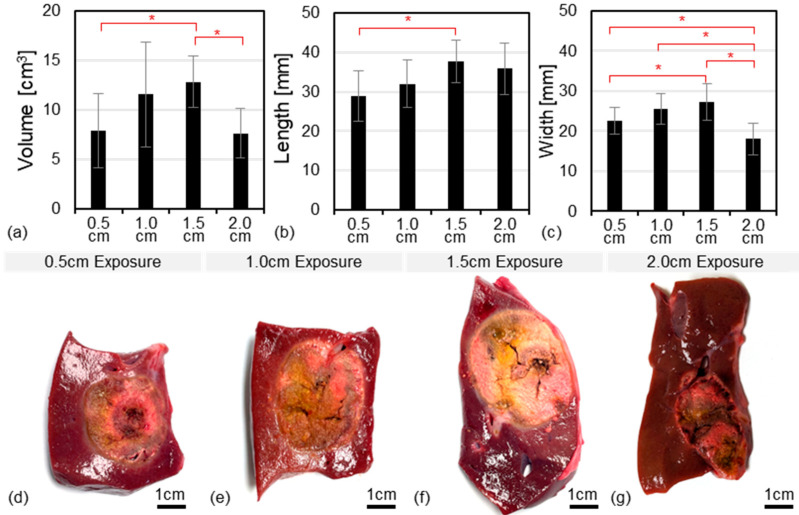
Rapid Production of Large Treatment Zones. Average (**a**) length, (**b**) width, and (**c**) volume of ablation zones as a function of electrode exposure length. Asterisks indicates values which were found to be significant different (*p* > 0.05). Error bars represent one standard deviation from the mean. Representative treatment zones created with (**d**) 0.5 cm, (**e**) 1.0 cm, (**f**) 1.5 cm, and (**g**) 2.0 cm electrode exposure lengths.

The ablation volumes measured 7.9 ± 3.7 cm^3^, 11.5 ± 5.3 cm^3^, 12.8 ± 2.6 cm^3^, and 7.6 ± 2.5 cm^3^ for the 0.5, 1.0, 1.5, and 2.0 cm exposed electrode lengths, respectively (Figure 8a). This corresponded to average measurements (length × width) of 2.8 × 2.2 cm, 3.2 × 2.5 cm, 3.7 × 2.7 cm, and 3.5 × 1.8 cm for the same respective groups (Figure 8b,c).

### 3.2. Numerical Modeling of Ablation Zones

**Figure 9:**
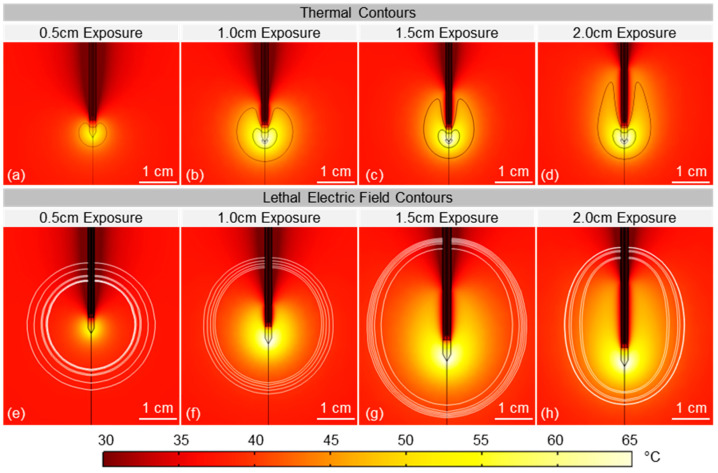
Thermal Transients During INSPIRE Treatment. Simulations of the temperature distribution at the end of treatment for electrode exposures of (**a**,**e**) 0.5 cm, (**b**,**f**) 1.0 cm, (**c**,**g**) 1.5 cm, and (**d**,**h**) 2.0 cm. Thermal iso-contours (black outlines in **a**–**d**) represent regions of 45 °C, 55 °C, and 65 °C predicted at the electrode tip. Electric field iso-contours (white outlines in **e**–**h**) corresponding to the calculated lethal threshold for each experimental treatment.

Time domain simulations were conducted to calculate the temperature distribution within the tissue and the electric field strength associated with the margin of each treatment (the lethal threshold) (Figure 9). These simulations predicted a cooled region along and directly adjacent to the applicator surrounded by regions of elevated temperature with the 45 °C isocontour extending approximately 1 cm into the tissue. Due to the electrode construction, the distal 0.5 cm tip of the electrode was not internally cooled. Therefore, the highest temperatures in the tissue occurred at the electrode tip which also corresponded to the region of highest electric field intensity. In all simulations, a region of tissue above 45 °C was predicted (Figure 9a–d). Smaller regions focused at the electrode tip of 55 °C and 65 °C were found for exposed electrode lengths of 1.0 cm and above (Figure 9c,d). The volume of tissue above 65 °C measured approximately 0, 0.02, 0.03, and 0.04 cm^3^ for 0.5 cm, 1.0 cm, 1.5 cm and 2.0 cm exposures, respectively. The volume of tissue above 45 °C measured approximately 0.17, 1.64, 2.76, and 3.92 cm^3^ for 0.5 cm, 1.0 cm, 1.5 cm and 2.0 cm exposures, respectively. Matching the ablation volumes to electric field intensity yielded calculated lethal electric field thresholds of 433 ± 102 V/cm, 548 ± 70 V/cm, 581 ± 63 V/cm, and 918 ± 183 V/cm (Figure 6).

## 4. Discussion

This study found that INSPIRE treatments can be safely administered with voltages up to 6000 V while using a single applicator and grounding pad approach. All treatments were completed successfully and no adverse events occurred intraoperatively or within the one week follow up period. The temperature control algorithm, which has previously been validated in in vitro [44], ex vivo [41], and in vivo [46] models, was able to achieve and maintain stable tissue temperatures throughout the treatments (Figure 3). Treatments were readily visible on contrast-enhanced CT and ultrasound imaging one week post treatment. Similarly, treatment zones were easily distinguishable from healthy liver parenchyma via palpation attributed to a marked increase in tissue stiffness in the treated region. Ablation volume increased sequentially as the exposed electrode length was increased from 0.5 to 1.5 cm followed by a significant (*p* < 0.5) decrease in volume for the 2.0 cm exposure group. The largest ablation volumes produced by the 1.5 cm exposure group measured 12.8 ± 2.6 cm^3^ corresponding to a 3.7 × 2.7 cm ablation zone created in approximately 5.2 min.

Temperature control during IRE treatments is a relatively new development [41,44,45]. While electroporation is generally considered a non-thermal phenomenon [47], application of electrical energy to tissue does induce a substantial thermal response via Joule heating which is proportional to the tissue conductivity and the applied electric field [48]. As tissue conductivity has a positive temperature coefficient, this can lead to a feed forward thermal response [48,49]. Therefore, significant tissue heating can occur with improper pulse parameter selection [50]. As treatment voltages are increased in search of larger treatment zones, thermal effects compound and can easily result in runaway tissue heating achieving temperatures of 100 °C or higher [41].

Active temperature control used in INSPIRE alleviates this challenge by dynamically adjusting energy delivery rates by increasing the delay between sequential waveforms as the temperature approaches the target setpoint. As the administered electrical dose is a critical factor in INSPIRE treatments [40], it is essential to deliver a sufficient number of pulses to achieve maximal ablation volumes. Therefore, the increase in delay between sequential waveforms necessarily increases overall treatment times [41]. Actively cooling the applicator can alleviate this challenge [51]. However, significant heating can still occur in this scenario with ex vivo studies indicating that 80 °C temperatures are readily achievable without temperature control [45]. This study indicates that the combination of electrode cooling and active temperature control is an effective strategy for reducing the potential for unintended thermal damage while maintaining clinically viable treatment times.

There are a number of factors that may affect INSPIRE treatment volumes including the pulse width [39], dose [46], voltage [39], and temperature [52,53]. In this study, these parameters were fixed to explore how the electrode exposure length affected treatment outcomes. In a 3D tumor model it was found that a 10× increase in NK-IRE dose, from 0.01 s to 0.1 s, resulted in a 6.8% increase in treatment size [52] while INSPIRE treatment sizes increased by 27 to 60% for this same 10× increase in dose [40,52]. In preliminary studies, we found that a dose of 0.02 s achieved the highest ablation efficiency, calculated as treatment volume/treatment duration. A dose of 0.02 s was therefore used in this study. Similarly, voltage was fixed to 6000 V as this is the maximum voltage achievable with our current prototype electronics.

The INSPIRE system used in this study was capable of producing alternating polarity waveforms with pulses between 250 ns and 100 µs. It has been shown in vitro [40] and in vivo [32] that treatment efficacy is reduced for pulses below 1000 ns. This corresponds to the approximate charging time of the cell membrane (400–1000 ns) [54]. Pulses below this characteristic timeframe generally require higher electric fields, therefore higher therapeutic voltages, to induce electroporation. Pulses longer than this characteristic timeframe generally reduce the electric field necessary to induce electroporation [55,56]; however, they also more easily stimulate muscle [57] and cardiac tissue [58]. Therefore, a pulse width of 1000 ns was selected in this study as a compromise between these phenomena. Higher doses, higher voltages, or longer pulse durations could be administered to increase the ablation volume at the expense of longer treatment durations and increased potential for muscle/cardiac stimulation. INSPIRE employs active temperature control, which limited treatment temperatures to 45 °C in this study. However, it will be of interest to examine how different temperature set points affect treatment outcomes.

This study examined only a single electrical dose (0.02 s); however, in vitro [40] and ex vivo [43] studies indicate that larger treatment zones may be possible with higher doses (0.04–0.1 s). As the protocols in this study required only 4 to 8 min to complete, larger ablation zones may be achievable in clinically relevant timeframes (e.g., less than 30 min) by administering higher doses. It will be of interest to investigate if ablation volumes can be meaningfully increased via this strategy. Alternatively, INSPIRE can be administered via multiple parallel electrodes akin to NK-IRE [59] and nsPEF procedures [38]. Ex vivo studies indicate that 4–5 cm ablation zones can be produced via this approach with INSPIRE type waveforms [43]; however, robust in vivo studies will be necessary to examine if similarly large treatment zones can be safely achieved in vivo.

With voltage, dose, pulse width, and temperature fixed to 6000 V, 0.02 s, 1000 ns and 45 °C, respectively, it was found that a 1.5 cm exposure length yields the largest and most consistent ablation volumes and this may be the optimal configuration for maximizing therapeutic efficiency (maximum volume per unit time). Variations in ablation volume with the 0.5 and 1.0 cm electrode exposures may have been due to subtle movement of the electrodes due to respiration which would have disproportionately affected the smaller electrode exposures. Of note, increasing the exposure length from 0.5 cm to 1.5 cm progressively increased the treatment volume at the expense of decreasing circularity of the treatment zone (length/width). Interestingly, an increase from 1.5 to 2.0 cm resulted in a reduction in overall treatment volume. In this single electrode and grounding pad configuration, the voltage in the tissue at the distal and proximal ends of the exposed electrode are equivalent. For long exposures this results in a large volume of tissue at the same electrical potential, thus reducing the overall electric field strength and reducing the volume of tissue exposed to lethal electric fields. Similar results were predicted for single insertion electrode arrays [60] and this study indicates that there is limited clinical utility for increasing electrode exposure length beyond 1.5 cm.

The electric field associated with treatment margins (the lethal threshold) varied significantly between exposed electrode lengths with a dramatic change between the 0.5 cm (433 V/cm) and 2.0 cm exposure (918 V/cm) configurations. These results are somewhat confounding as the pulse width (1000 ns), electrical dose (0.02 s) and voltage (6000 V) were constant across all treatment groups. Based on in vitro studies [40], this should have resulted in consistent lethal electric field values independent of the exposed electrode length. It has been shown in vitro that IRE is a thermally mediated phenomenon for pulses on the order of 1000 ns [53] and variations in tissue temperature distributions for each exposed electrode length may have affected the lethal threshold. Prior studies indicate that higher tissue temperatures should result in larger treatment zones [53,61,62]; however, the treatments in this study with the highest simulated temperatures (2.0 cm exposures) had the smallest overall treatment volumes and highest lethal thresholds. An alternative explanation may be that complex changes in tissue conductivity are occurring throughout the treatment due to heating and possible tissue desiccation that the numerical model fails to predict. Tissue conductivity has a strong temperature dependence which may lead to substantial changes in the conductivity adjacent to the electrode. While this was simulated as a linear change proportional to temperature, a more complex non-linear process (e.g., tissue desiccation) may be dominating experimentally, resulting in improper calculation of the electric field distribution around the electrode and leading to the observed variability in lethal electric field calculations between treatment groups. Until this is better understood, caution should be used when using the values predicted here for the development of new electrodes or for treatment planning with electrode geometries that are different than those presented here.

There is the potential for intense muscle stimulation [24] and the serious cardiac complications with NK-IRE [21,22,63]. This is associated with inadvertent activation of these tissues by the relatively long monopolar (80–100 µs) electrical pulses used which can stimulate muscle (1.8 V/cm) [57] and cardiac tissue (5.5 V/cm) [58] at low electric field strengths. These low stimulatory field strengths result in a large area around the therapeutic electrodes (effectively the entire body) which is subject to potential stimulation. In contrast, substantially higher field strengths are required to stimulate muscle (112 V/cm) [57] and cardiac (195 V/cm) [58] tissue with the 1000 ns pulses used in this study. This dramatically reduces the regions of tissue subject to stimulatory field strengths to areas directly adjacent to the applicator. While NK-IRE requires cardiac synchronization [21,63,64], this study indicates that INSPIRE treatments can be safely administered without active synchronization. The average pre- and post-treatment troponin levels were 0.249 ng/mL and 0.224 ng/mL, respectively. Post-INSPIRE levels were substantially below those associated sham cardiac surgical procedures (2 ng/mL) [65], non-necrosing cardiac ischemia (42 ng/mL) [66], necrosing cardiac ischemia (127 ng/mL) [66] and myocardial infarction in conjunction with cardiac arrest (88 ng/mL) [65] in pigs. While conclusive clinical studies will be necessary, these results indicate that INSPIRE may offer an improved safety profile over NK-IRE.

Treatment zones following INSPIRE were visible on ultrasound in the intraoperative period with contrast improving approximately 5 to 25 min after completion (Figure 4) indicating that it may be possible to confirm treatment success for this modality via ultrasound. However, comprehensive studies comparing intraoperative ultrasound and gross pathology will be necessary to confirm the sensitivity of these measurements and their utility in predicting therapeutic outcomes. While real time visualization of treatments is possible with NK-IRE in healthy liver parenchyma [67], the sensitivity of measurements is widely ranging and ultrasound has failed to establish itself as a reliable method for treatment confirmation [4,68,69]. It is possible that the ultrasound contrast observed in healthy liver parenchyma following INSPIRE may be muted in tumor tissue where the tissue structure is less well organized. The treatment sites were clearly visible on contrast-enhanced CT one week following treatment (Figure 5a) and we observed similar contrast in INSPIRE-treated veterinary liver tumors indicating that CT is promising imaging modality for ablation assessment. It will be of interest to examine the time course development of CT contrast following INSPIRE to assess if intraoperative CT imaging can supplement ultrasound imaging as a means of determining treatment success.

## 5. Conclusions

This study demonstrates the in vivo safety and feasibility of delivering high-voltage (6000 V) INSPIRE treatments using a single applicator and grounding pad configuration. Simulations indicate that the system’s integrated temperature control algorithm effectively maintained bulk tissue temperatures below thermal injury thresholds while allowing the delivery of ablative electrical doses. Treatment volume was strongly influenced by exposed electrode length, with a 1.5 cm electrode producing the largest and most consistent ablations. INSPIRE achieved large ablation volumes without the need for multiple electrode placements or cardiac synchronization. Troponin analysis confirmed minimal cardiac impact, supporting a favorable safety profile. Together, these findings suggest that INSPIRE enables efficient, reproducible, and non-thermal ablation with reduced procedural complexity compared to NK-IRE. Studies to assess anti-tumor efficacy, long-term tissue response, and clinical translation are warranted.

## Figures and Tables

**Table 1:** Study parameters and resulting treatment times and volumes.

Electrode Length [cm]	Replicates [N]	Treatment Time [s]	Volume [cm^3^]
0.5	9	473 ± 83	7.95 ± 3.74
1.0	6	421 ± 91	11.56 ± 5.32
1.5	7	314 ± 63	12.83 ± 2.61
2.0	5	250 ± 105	7.64 ± 2.50

## Data Availability

The original contributions presented in this study are included in the article. Further inquiries can be directed to the corresponding author.

## Abbreviations

The following abbreviations are used in this manuscript:

INSPIRE	Integrated Nanosecond Pulsed Irreversible Electroporation
IRE	Irreversible Electroporation
NK-IRE	NanoKnife Irreversible Electroporation
PFA	Pulsed Field Ablation
PEF	Pulsed Electric Field
RF	Radiofrequency Ablation
MWA	Microwave Ablation
CA	Cryoablation
CT	Computed Tomography
